# A high-content phenotypic screen identifies luteolin as a repurposed drug to reduce pyruvate dehydrogenase phosphorylation and enhance energy production in CMTX6 cell models

**DOI:** 10.1093/jmcb/mjag012

**Published:** 2026-03-23

**Authors:** Gonzalo Perez-Siles, Masahiro Nishide, Melina Ellis, Richard M Wynn, Gauri Shishodia, Steve Vucic, Marina L Kennerson

**Affiliations:** School of Medical Sciences, Faculty of Medicine and Health, University of Sydney, Sydney, NSW 2050, Australia; ANZAC Research Institute, Sydney Local Health District, Concord, NSW 2139, Australia; ANZAC Research Institute, Sydney Local Health District, Concord, NSW 2139, Australia; ANZAC Research Institute, Sydney Local Health District, Concord, NSW 2139, Australia; Department of Biochemistry, University of Texas Southwestern Medical Center, Dallas, TX 75390-9038, USA; Department of Biochemistry, University of Texas Southwestern Medical Center, Dallas, TX 75390-9038, USA; Brain and Nerve Research Centre, Concord Repatriation General Hospital, Concord, NSW 2139, Australia; School of Medical Sciences, Faculty of Medicine and Health, University of Sydney, Sydney, NSW 2050, Australia; ANZAC Research Institute, Sydney Local Health District, Concord, NSW 2139, Australia; Molecular Medicine Laboratory, Concord Repatriation General Hospital, Sydney, NSW 2139, Australia

**Dear Editor**,

Charcot–Marie–Tooth disease type 6 (CMTX6) is a rare X-linked inherited neuropathy caused by missense mutations (p.R158H and p.R162H) in the pyruvate dehydrogenase kinase 3 (*PDK3*) gene (Kennerson et al., 2013). These mutations cause constitutive activation of PDK3 (Supplementary Figure S1), resulting in excessive phosphorylation of the Ser293 and Ser300 residues in the E1α subunit of the pyruvate dehydrogenase complex (PDC) in patient-derived fibroblasts (Perez-Siles et al., 2016), the inhibition of mitochondrial oxidative metabolism in induced pluripotent stem cell (iPSC)-derived motor neurons (Perez-Siles et al., 2020), and locomotion defects in a *Caenorhabditis elegans* CMTX6 model (Narayanan et al., 2021). Despite recent advances in understanding the molecular mechanisms underlying CMTX6, no disease-modifying therapies are currently available for this disease.

To identify compounds capable of reversing the biochemical signature of CMTX6, we developed a high-content phenotypic screen (HCS) using fibroblasts derived from a male patient carrying the p.R158H PDK3 mutation. These cells show elevated mitochondrial PDC-E1α phosphorylation and provide a robust and scalable readout for quantifying mitochondrial dysfunction in CMTX6. Using immunofluorescence and automated image analysis, we screened 1840 US Food and Drug Administration (FDA)-approved compounds (MedChemExpress). MitoTracker and nuclear staining were combined with immunolabeling for pSer293, the most functionally relevant Ser residue with the highest catalytic efficiency for PDK3-mediated phosphorylation (Korotchkina and Patel, 2001), to enable accurate segmentation and quantification of mitochondrial PDC phosphorylation. For each well, Z-scores were calculated relative to untreated patient fibroblasts (Z-score = 0) and control fibroblasts (Z-score = −5). Dichloroacetic acid (DCA), a broad-spectrum PDK inhibitor known to restore PDC-E1α phosphorylation, was included as a positive control (Z-score = −4) for drug treatment effectiveness. This assay achieved a Z’ factor of 0.32, indicating acceptable reproducibility and robustness for the HCS.

The primary screen was performed at a single dose of 10 μM in a single replicate (Figure 1B), identified 48 compounds with Z-scores below −4 (predefined threshold for compound effectiveness), which were then re-tested in replicate plates (Figure 1C). Subsequently, 24 compounds with reproducible effects were evaluated in dose–response assays across a 0.01–50 μM concentration range (Figure 1D). This confirmed 12 small molecules that could reduce PDC-E1α phosphorylation in CMTX6 patient-derived fibroblasts in a dose-dependent manner. We then conducted a secondary screen in iPSC-derived motor neurons generated from the same CMTX6 patient. These motor neurons (MN^CMTX6^) recapitulate the fibroblast metabolic phenotype and display reduced energy levels associated with impaired mitochondrial respiration compared with isogenic controls (Perez-Siles et al., 2020). ATP levels (Figure 1E) and cell viability (Supplementary Figure S2) were measured in parallel to exclude cytotoxic compounds. Four FDA-approved small molecules (sacubitril, mebhydroline, nitrofurazone, and luteolin) emerged as positive hits that significantly increased ATP production after 72 h treatment while maintaining neuronal integrity. Among these, luteolin was most effective in restoring bioenergetic capacity in MN^CMTX6^ without affecting cell viability. Immunofluorescence of neuronal cultures confirmed a reduction in pSer293 signal intensity following treatment of all four positive hits (Figure 1F; Supplementary Figure S3), indicating an engagement of the PDC pathway in spinal motor neurons.

**Figure 1:**
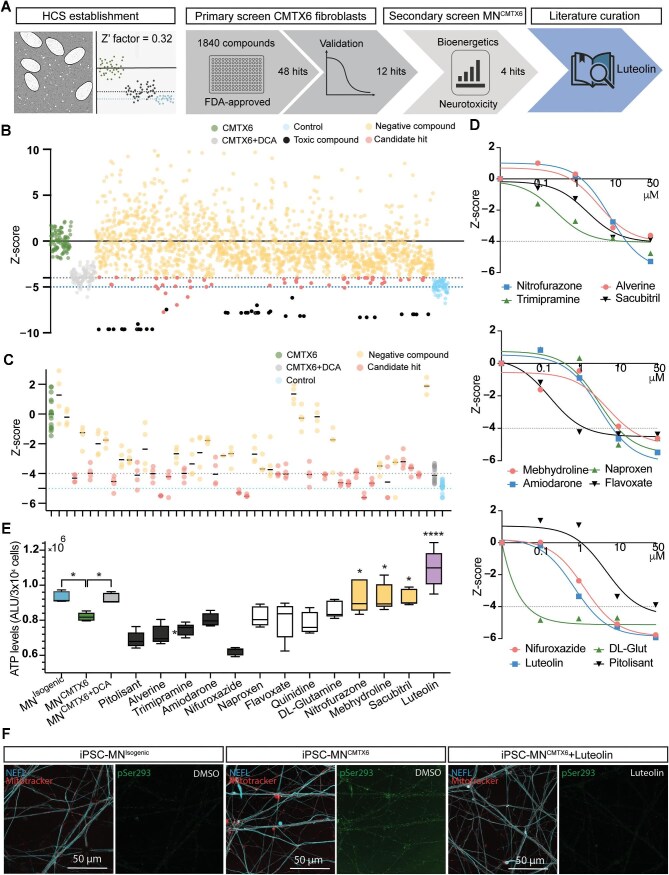
HCS identifies luteolin as a top FDA-approved candidate to reduce PDC-E1α phosphorylation and enhance energy production in CMTX6 cell models. (**A**) Primary screening was performed in CMTX6 patient fibroblasts, followed by secondary screening in iPSC-derived motor neurons. Final candidate prioritization was informed by literature curation. (**B**) Phenotypic assay in CMTX6 fibroblasts using differential immunofluorescence staining yielding robust Z-scores across experimental groups (Z’ factor = 0.32). CMTX6 cells (5 × 10^3^cells/well) were plated in 384-well glass-bottom plates and treated with 1840 FDA-approved compounds (10 μM; MedChemExpress, HY-L022M) in 0.1% (*v*/*v*) dimethyl sulfoxide (DMSO) for 24 h (yellow dots). Control fibroblasts from a healthy donor treated with 0.1% (*v*/*v*) DMSO defined physiological PDC-E1α phosphorylation levels (blue dots), while 5 mM DCA-treated CMTX6 fibroblasts served as positive controls (grey dots). Data were normalized to 0.1% (*v*/*v*) DMSO (vehicle)-treated CMTX6 fibroblasts (green dots). PDC-E1α phosphorylation was quantified using anti-pSer293 antibody (Millipore, AP1062, 1:250). Automated confocal imaging (Leica SP8, 10 fields/condition) and an in-house CellProfiler developed pipeline was used to determine the Z-score. Positive hits were defined as the compounds with Z-scores < −4. (**C**) Forty-eight primary hits were re-tested at 10 μM in quadruplicate, confirming 24 compounds that consistently reduced PDC-E1α phosphorylation (red dots, Z-scores < −4). (**D**) Twelve candidate compounds underwent further validation by dose–response testing (0.1–50 μM) in triplicate to assess efficacy and potency. (**E**) Secondary screening was performed in MN^CMTX6^ (3 × 10^4^ cells/well in 96-well matrigel-coated plate; 8 wells per drug) by assessing ATP production. At 32 days *in vitro* (DIV), cells were treated for 72 h with the compounds selected from the primary screen. ATP levels were measured at 35 DIV using the ATPlite kit (PerkinElmer). Sacubitril, mebhydroline, nitrofurazone (yellow), and luteolin (magenta) significantly increased ATP levels relative to that in untreated MN^CMTX6^ (green). Initial candidates that retrieved no significant changes in ATP levels are shown in white. Data are presented as arbitrary luminescence units (ALU); two-way analysis of variance with Tukey’s *post hoc* multiple comparations test (*P < 0.05; ****P < 0.0001). Cytotoxicity of identified compounds (black) was assessed using the CCK-8 kit (Sigma-Aldrich). (**F**) Representative immunofluorescence images showing PDC-E1α phosphorylation (pSer293, green) in MN^CMTX6^ (NEFL, light blue; mitochondria, red) following 72 h treatment with 10 μM luteolin or 0.1% (*v*/*v*) DMSO and the isogenic control (iPSC-MN^Isogenic^).

Finally, a literature-based prioritization of the screening hits was performed (Supplementary Table S1). Scientific evidence advised against nitrofurazone, an antimicrobial organic compound, due to reported *in vivo* toxicity and carcinogenicity (Kari et al., 1989). Sacubitril, a neprilysin inhibitor, in combination with valsartan (SAC/VAL) is an FDA-approved medication for chronic heart failure. SAC/VAL administration has also been reported to confer neuroprotection in models of Parkinson’s disease-associated neurodegeneration, and neprilysin inhibitors have been proposed to exert protective effects in Alzheimer’s disease (Nalivaeva et al., 2020). However, the reported mechanism of sacubitril is associated with upregulation of WNT/β-catenin signaling, and no direct link to pyruvate dehydrogenase function has been established. The available literature for mebhydroline (a first-generation antihistamine) provides little evidence for a role in mitochondrial metabolism, and its effects on neuronal function or health have not been reported.

Of the compounds identified from our phenotypic drug screening, luteolin emerged as the most effective one for increasing ATP production in MN^CMTX6^ (Figure 1E). Luteolin is a naturally occurring flavonoid with well-described anti-inflammatory, antioxidant, and neuroprotective properties. Accumulating evidence supports luteolin’s protective effect on several *in vitro* and *in vivo* neurodegenerative disease models (Jayawickreme et al., 2024). Notably, a recent neuronal cell-based high-throughput screen identified luteolin as an enhancer of mitochondrial function, improving mitochondrial respiration (Naia et al., 2021). Although a direct effect of luteolin on PDK3 activity or PDK3 protein abundance in MN^CMTX6^ cannot be excluded, the findings by Naia et al. (2021) suggest an alternative mechanism whereby luteolin modulates mitochondria–ER interactions, promoting calcium release from the ER and subsequent activation of pyruvate dehydrogenase phosphatases. This pathway provides a plausible explanation for the reduced PDC-E1α phosphorylation following luteolin treatment in MN^CMTX6^; however, its relevance in the CMTX6 setting remains speculative and will require targeted experimental validation in our model.

This study underscores the utility of phenotypic screening in patient-derived fibroblasts for identifying candidate therapies in rare diseases. High-content imaging coupled with quantitative analysis enabled the robust, scalable detection of subtle changes in mitochondrial function. Cross-validation in patient iPSC-derived neurons further strengthened the disease relevance of our findings. In summary, we show that luteolin significantly reduces PDC-E1α phosphorylation and restores ATP production in cell models of CMTX6, highlighting luteolin as a lead compound for further preclinical evaluation in CMTX6. While several studies report no evidence of neurotoxicity and suggest an overall favorable safety profile for luteolin (Naiki et al., 2025), *in vivo* testing in CMTX6 models will be essential to confirm its efficacy, safety, and therapeutic potential in this specific disease context. In parallel, further mechanistic studies in MN^CMTX6^, interrogating additional disease-relevant phenotypes, are needed to define luteolin’s mode of action and to substantiate its therapeutic promise for CMTX6 patients.

*[Supplementary material is available at *Journal of Molecular Cell Biology* online. We thank the families who have participated in our research. The work has been funded by NHMRC Ideas Grant (APP11868687 awarded to M.L.K. and G.P.-S.) and Medical Research Future Fund Grant (MRFF 2007681 awarded to M.L.K., S.V., and G.P.-S).].*

## Supplementary Material

mjag012_Supplemental_File
