# Supplementary material for: A high-content phenotypic screen identifies luteolin as a repurposed drug to reduce pyruvate dehydrogenase phosphorylation and enhance energy production in CMTX6 cell models
Source: J Mol Cell Biol. 2026 Mar 23;18:mjag012. doi: 10.1093/jmcb/mjag012 (PMC13344419; doi:10.1093/jmcb/mjag012)
Supplement: mjag012_Supplemental_File [file mjag012_supplemental_file.pdf]

Supplementary Figures

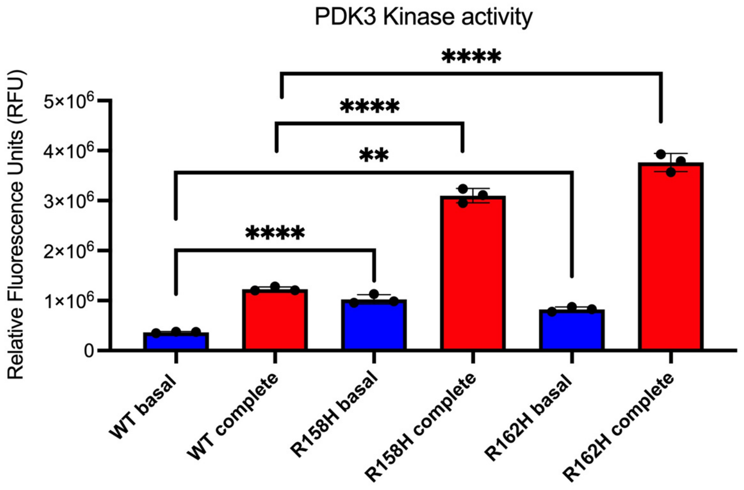

Supplementary Figure S1 CMTX6-associated PDK3 missense mutations (p.R158H and p.R162H) exhibit PDK3 kinase hyperactivity as a shared disease mechanism. Enzymatic activity of wild-type (WT) and mutant PDK3 (p.R158H or p.R162H) was assayed using the E1p of the pyruvate dehydrogenase complex (PDC) as substrate following previously described protocols (Kennerson et al., 2013). Recombinant human PDK3 proteins N-terminally fused to maltose-binding protein (MBP-PDK3) were expressed and purified. Kinase activity was measured under two conditions: basal activity in the absence of the E2p/E3BP core of PDC (blue) and activity in the presence of the E2p/E3BP core to assess co-factor enhanced stimulation (red). Data were obtained from three independent experiments and presented as mean ± standard error of the mean (SEM). Two-way ANOVA with Tukey's *post hoc* multiple comparations test (**$P < 0.005$; ****$P < 0.0001$).

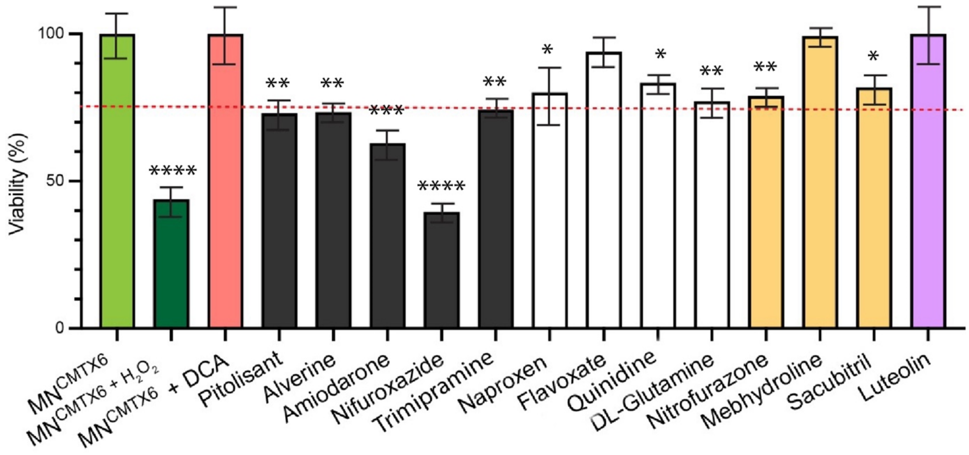

Supplementary Figure S2 Cytotoxicity profiling of candidate compounds in CMTX6 iPSC-derived motor neurons using the CCK-8 assay. Differentiated CMTX6 iPSC-derived motor neurons were treated with selected candidate compounds (10 μM) for 72 h. Cell viability was assessed using the CCK-8 assay and expressed as a percentage of untreated controls. Hydrogen peroxide (H_2O_2, 200 μM) served as a positive control for cytotoxicity. The red dotted line indicates a 75% viability threshold, applied here as a conservative

cut-off to exclude potentially cytotoxic hits. This threshold was set arbitrarily to prioritize compounds with minimal adverse effects on cell viability (Inglese et al., 2007). Data are presented as mean ± SEM from three independent replicates. Two-way ANOVA with Tukey's post hoc multiple comparations test (*$P < 0.05$; **$P < 0.005$; ***$P = 0.0001$; ****$P < 0.0001$). Luteolin, sacubitril, mebhydroline, and nitrofurazone were considered for further validation.

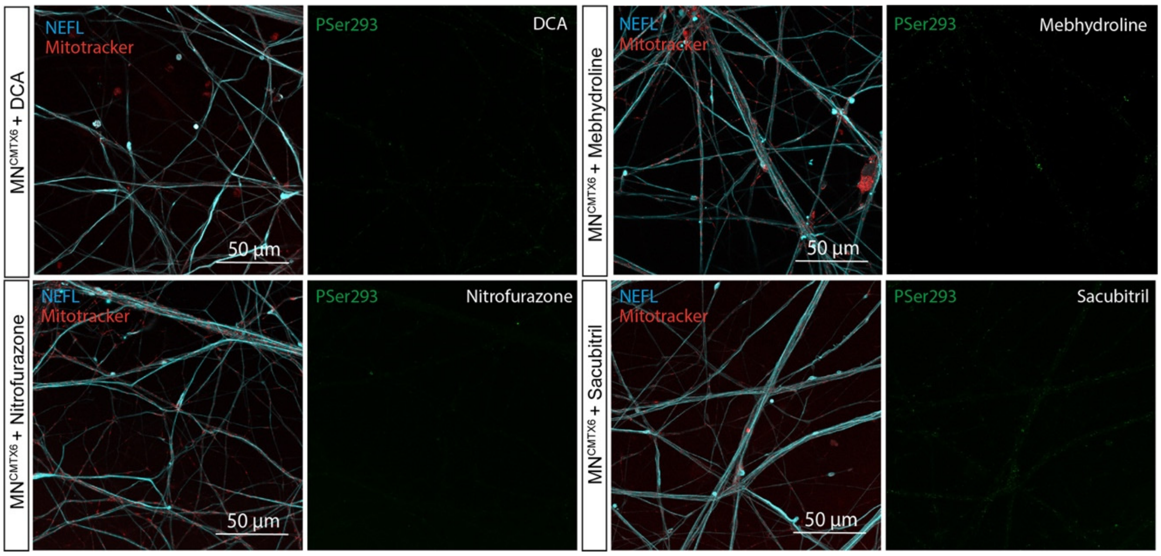

Supplementary Figure S3 Immunofluorescence analysis confirms the reduced PDC-E1α hyperphosphorylation in the treated CMTX6 iPSC-derived motor neurons (MN^{CMTX6}). MN^{CMTX6} were treated with 10 µM sacubitril, mebhydroline, or nitrofurazone or 2.5 µM DCA for 72 h. Immunofluorescence staining revealed the reduction in PDC-E1α phosphorylation (green) following treatment. MN^{CMTX6} were co-stained with NEFL (light blue) and a mitochondrial marker (mitotracker; red).

Supplementary References

Inglese, J., Johnson, R.L., Simeonov, A., et al. (2007). High-throughput screening assays for the identification of chemical probes. Nat. Chem. Biol. *3*, 466–479. doi: 10.1038/nchembio.2007.17. PMID: 17637779.

Kennerson, M.L., Yiu, E.M., Chuang, D.T., et al. (2013). A new locus for X-linked dominant Charcot–Marie–Tooth disease (CMTX6) is caused by mutations in the pyruvate dehydrogenase kinase isoenzyme 3 (PDK3) gene. Hum. Mol. Genet. *22*, 1404–1416. doi: 10.1093/hmg/dds557. PMID: 23297365; PMCID: PMC3596851.

Supplementary Table S1 Literature-based curation of the top four candidate compounds identified from the phenotypic high-content screen.

**Compound/PubChem CID**	**Drug class**	**Known main mechanistic action**	**Affected cellular pathway**	**References (PMID)**
Sacubitril/9811834	Neprilysin inhibitor (prodrug)	WNT/β-catenin signaling, promoting Drp1 dephosphorylation	Activates natriuretic peptides and enhances mitochondrial respiration	28623750; 37099206; 38341600; 41138763
Mebhydroline/22530	First-generation H1 antihistamine	Farnesoid X receptor antagonist	Suppresses hepatic gluconeogenesis and promotes glycogen synthesis	33831422
Nitrofurazone/5447130	Antibacterial (nitrofuran family)	Inhibits bacterial enzymes, disrupting DNA synthesis	Induces oxidative stress and mitochondrial damage	2714718; 3179282; 41076910; 15488632;
Luteolin/5280445	Flavonoid/polyphenol	Increases mitochondria–ER interaction, increasing mitochondrial calcium and activating pyruvate dehydrogenase phosphatases	Reduces oxidative stress and enhances mitochondrial respiration and ATP production.	33761951; 38904713; 40154935; 39395694

This table summarises the key pharmacological and mechanistic properties of the top four hit compounds including their drug class, primary mode of action, associated cellular pathways, and relevant supporting literature (PubMed PMIDs). Nitrofurazone (red) has been de-prioritised due to reported toxicity and carcinogenicity. Luteolin (green) has emerged as the most promising candidate, based on its efficacy across assays and strong prior evidence supporting its mitochondrial and neuroprotective effects, making it a lead compound for further clinical investigation in CMTX6.
